# A Quasi In-Situ Study on the Microstructural Evolution of 2195 Al-Cu-Li Alloy during Homogenization

**DOI:** 10.3390/ma15196573

**Published:** 2022-09-22

**Authors:** Hao Huang, Wei Xiong, Zhen Jiang, Jin Zhang

**Affiliations:** 1 Light Alloy Research Institute, Central South University, Changsha 410083, China; 2 State Key Laboratory of High Performance and Complex Manufacturing, Central South University, Changsha 410083, China

**Keywords:** Al-Cu-Li alloy, homogenization, microstructural evolution, quasi in situ

## Abstract

An optimized homogenization process for Al alloy ingots is key to subsequent material manufacturing, as it largely reduces metallurgical defects, such as segregation and secondary phases. However, studies on their exact microstructural evolution at different homogenization temperatures are scarce, especially for complex systems, such as the 2195 Al-Cu-Li alloy. The present work aims to elucidate the microstructural evolution of the 2195 Al-Cu-Li alloy during homogenization, including the dissolution and precipitation behavior of the T_B_ (Al_7_Cu_4_Li) phase and S (Al_2_CuMg) phase at different homogenization temperatures. The results show that there are Cu segregation zones (Cu-SZ) at the dendrite boundaries with θ (Al_2_Cu) and S eutectic phases. When the temperature rises from 300 °C to 400 °C, fine T_B_ phases precipitate at the Cu-SZ, and the Mg and Ag in the S phases gradually diffuse into the matrix. Upon further increasing the temperature to 450 °C, T_B_ and θ phases at the grain boundaries are coarsened, and an S-θ phase transition is observed. Finally, at 500 °C, all T_B_ and S phases are dissolved, leaving only θ phases at triangular grain boundaries. This work provides guidance for optimizing the homogenization procedure in 2195 alloys.

## 1. Introduction

Al-Cu-Li alloys possess higher specific strength and specific stiffness compared with traditional aluminum alloys, enabling considerable weight reductions while maintaining good mechanical properties in aerospace applications [1,2,3,4,5,6]. They are considered to be desirable structural parts for the aerospace industry now [7,8,9,10,11,12]. However, existing studies show that large structural portions of Al-Cu-Li alloys are inclined to cracking during severe deformation and, therefore, have poor formability [13,14]. To solve these problems, cast ingots of Al-Cu-Li alloys are usually homogenized prior to manufacturing. The microstructural evolution of these ingots during homogenization has a significant impact on their deformation, recovery and recrystallization behaviors in subsequent processes, such as rolling and forging [15,16,17,18,19,20]. For example, T_B_ and θ phases on the grain boundaries will dissolve in the appropriate homogenization process, thus inhibiting the initiation of cracks during subsequent processing [21,22] and improving their mechanical properties after aging [23,24,25]. Therefore, an in-depth investigation of the microstructural evolution during the homogenization of Al-Cu-Li alloys is significant for understanding their material properties and optimizing subsequent processing. The dissolution behavior of T_B_ and S phases especially needs to be elucidated as these eutectic phases commonly exist in Al-Cu-Li alloys, and their distribution at grain boundaries severely deteriorates the formability of the materials.

Liu et al. [26] researched the impacts of homogenization on the T_B_, θ and S phases in Al-3.8Cu-1.28Li-0.4Mg alloy. It was found these eutectic phases are completely dissolved at 530 °C with extended homogenization time. Li et al. [27,28] reported that T_B_ and S phases are only partially dissolved after the first-step homogenization at 400 °C for 8 h, and their complete dissolution occurs during second-step homogenization. Liu and Li only studied the dissolution of primary eutectic T_B_ and S phases in the ingot during homogenization; they did not notice that the T_B_ phase will also precipitate during homogenization and that the S phase will transform into the θ phase. Yang et al. [29] found that there are plenty of θ phases and little of S phases on the grain boundary of the Al-3.52Cu-1.28Li-0.38Mg ingot, and the S phases are preferentially dissolved during homogenization as their melting point is lower. Although Yang thought that the S phase dissolves first compared to the θ phase, he did not find that the S phase is first transformed into the θ phase and then dissolved. Chen et al. [30] studied the Al-5Cu-1Li-0.6Mg alloy and observed that θ and T_1_ phases are coarsened during homogenization, and T_B_ and θ phases are dissolved at elevated temperatures. Chen noticed that the T_B_ phase coarsened during homogenization, but he did not find that the T_B_ phase will precipitate during homogenization and did not distinguish the primary eutectic T_B_ phase from the T_B_ phase that precipitated later. The above studies on the microstructural evolution in the homogenization of Al-Cu-Li alloys are not comprehensive enough. To date, there has been little research on the detailed precipitation and dissolution behavior of T_B_ and S phases during homogenization, such as the S phase transforming into the θ phase and the newly precipitated T_B_ phase during homogenization.

Some researchers have studied the nucleation and growing of second phase particles during the cooling of homogenized aluminum alloy, the micro-segregation of alloying elements during the homogenization of aluminum alloy, and stress analysis by computational simulation methods [31,32,33]. In this study, the microstructural evolution of the 2195 Al-Cu-Li alloy during hominization is obtained via direct experimental observations, which is facile and commonly adopted by the research community, this could provide an experimental basis, such as boundaries conditions for subsequent computational simulation research.

To more intuitively investigate the precipitation and dissolution behavior of T_B_ and S phases, a combination of methods, including quasi in situ scanning electron microscopy (SEM), transmission electron microscopy (TEM), X-ray diffraction (XRD), Vickers hardness tests and energy-dispersive X-ray spectroscopy (EDS), were applied to study the phase composition, microstructure and elemental distribution of the 2195 alloy at different homogenization temperatures (25–500 °C). We demonstrate that, by raising the homogenization temperature from 300 °C to 450 °C, a fine T_B_ phase forms by precipitating from Cu segregation zones and then coarsens; these are continuously distributed on the grain boundaries, while the S phase first transforms into the θ phase and then dissolves. These results can be used to optimize the homogenization procedure for 2195 alloys.

## 2. Materials and Methods

The experimental material employed is an Al-Cu-Li alloy ingot made by the central south university (Ø180 mm × 500 mm). The elemental ingredients were verified by the inductively coupled plasma-atomic emission spectroscopy (ICP-AES) (SPECTRO BLUE SOP, Kleve, Germany) (see Table 1).

**Figure 1:**
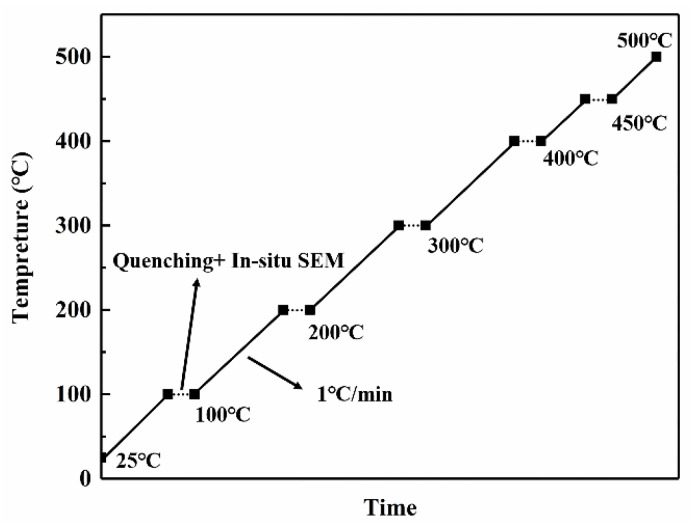
Schematic diagram of homogenization procedure.

**Figure 2:**
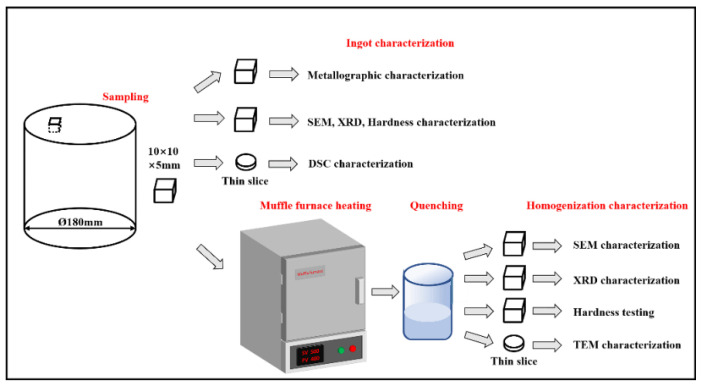
Schematic diagram of the workflow.

A 10 × 10 × 5 mm sample was taken from the ingot. We cut a small-sized sample to reduce the temperature difference between the interior and exterior of the sample during homogenization. The sample was put into the muffle furnace (SG-XS1700, Shanghai Sager Furnace Co., Limited, Shanghai, China) and heated from 25 °C to 500 °C; the heating rate of the muffle furnace was 1 °C/min and the temperature error was controlled within ±1 °C. The sample was quenched with water (less than 5 s) after reaching 100 °C, 200 °C, 300 °C, 400 °C, 450 °C and 500 °C, followed by quasi in situ SEM observations. The observation area was located by Vickers hardness indentation on the sample. The temperature of the furnace was maintained until the sample was put back after finishing quasi in situ SEM observation. The homogenization procedure is presented in Figure 1, and the workflow diagram is displayed in Figure 2.

X-ray diffraction (XRD) (D/Max 2550VB, Rigaku, Tokyo, Japan) was used to identify the secondary phases, the scanning rate was 4 °/min and 2θ has a range of 20° to 100°. The power of the X-ray generator is 18 kW and the stability error of the X-ray generator is within ±0.01%. The Differential Scanning Calorimetry (DSC) (DSC8000, PerkinElmer, Waltham, MA, USA) was applied to verify the non-equilibrium solidus temperature of the ingot by heating the sample from 0 to 600 °C at 10 k/min. The precipitation behavior was detected using transmission electron microscopy (TEM) (Tecnai G2F20, FEI, Lincoln, NE, USA). The acceleration voltage was 200 KV. A Vickers hardness tester (YZHV-1000P, Yizong Precision Instrument Co., Ltd., Shanghai, China) was applied to test the hardness of the sample.

The evolution of the microstructure during homogenization was investigated with a Scanning Electron Microscopy (SEM) (EVO MA10, ZEISS, Oberkochen, Germany) instrument possessing an Energy Dispersive Spectrometer (EDS) detector; the accelerating voltage is 20 kV, and the elemental distribution was studied using the EDS line-scanning (LS) and map-scanning (MS) modes. The SEM resolution was 3.0 nm.

## 3. Results and Discussion

### 3.1. Microstructural Characterization of 2195 Ingot

**Figure 3:**
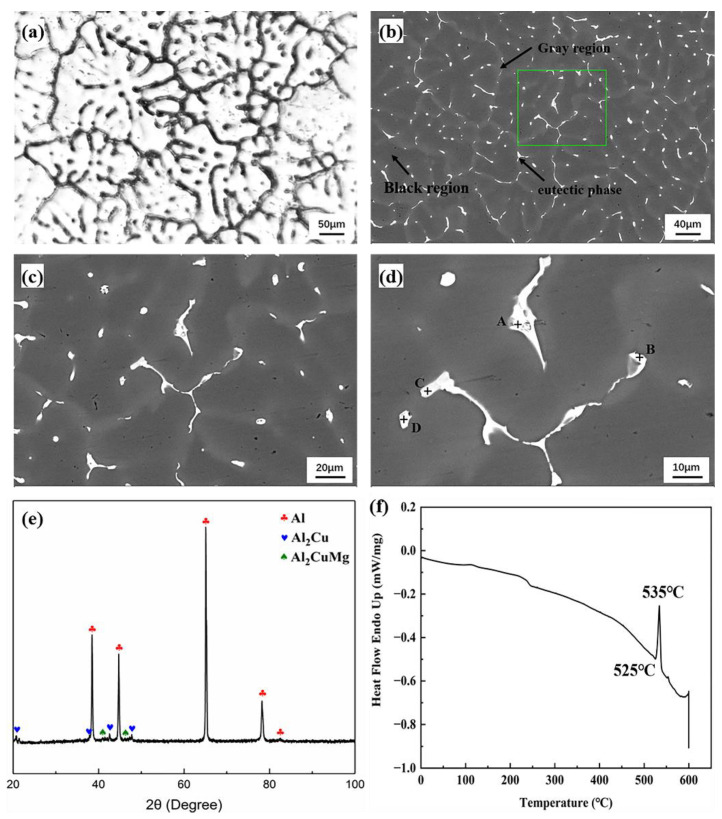
2195 ingot microstructure: (**a**) ingot metallographic image; (**b**) in situ observation zone; (**c**,**d**) 500× and 1000× magnification of in situ observation zone; (**e**) ingot XRD; (**f**) ingot DSC.

**Figure 4:**
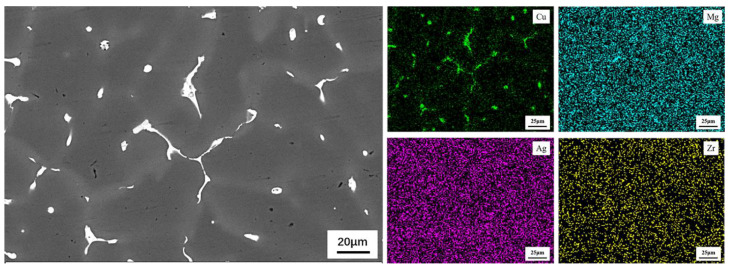
EDS elemental distribution of QISOZ.

Figure 3a displays the metallographic image of the 2195 ingot, where obvious dendritic structures are observed, and the mean grain size is 150–250 μm. Figure 3b displays the backscattered SEM image of the ingot, and the boxed area in the Figure is the quasi in situ observation zone (QISOZ). As shown by the arrows, there is a high number of gray regions (these regions were proved to be Cu segregation zones in Figure 4); the non-equilibrium eutectic phases are distributed in the gray regions. The size and shape of these gray regions are similar to the corroded zones in the metallographic image. Figure 3c,d show the QISOZ backscattered SEM images at 500× and 1000× magnification, respectively. The EDS results of each point in Figure 3d are shown in Table 2. There are θ and S phases in the ingot, and a certain amount of Ag exists in the S phase. This is because the interactive energy between Ag and Mg atoms was strong (−0.0632 eV) [34]; hence, Ag is easily combined with Mg to form Mg-Ag clusters [35,36,37,38,39]. Figure 3e presents the XRD result of 2195 ingot. Besides α(Al), θ phases and S phases also exist in the ingot, which is identical to the EDS results in Figure 3d. Figure 3f displays the DSC result of the ingot. There is an apparent endothermic peak at 525 °C in the ingot, which led to the melting of some low-melting-point eutectic phases, indicating that the non-equilibrium solidus temperature of the ingot is 525 °C. Therefore, in this study, the maximum temperature of the homogenization was chosen to be 500 °C to avoid over-burning the sample.

The EDS elemental distribution of Cu, Mg, Ag and Zr on the QISOZ is displayed in Figure 4. Cu is segregated in the gray regions, so the gray regions are named Cu segregation zones (Cu-SZ). Mg, Ag and Zr were homogeneously distributed, while Cu shows severe segregation.

### 3.2. Microstructural Characterization of the Sample at 100 °C, 200 °C and 300 °C

**Figure 5:**
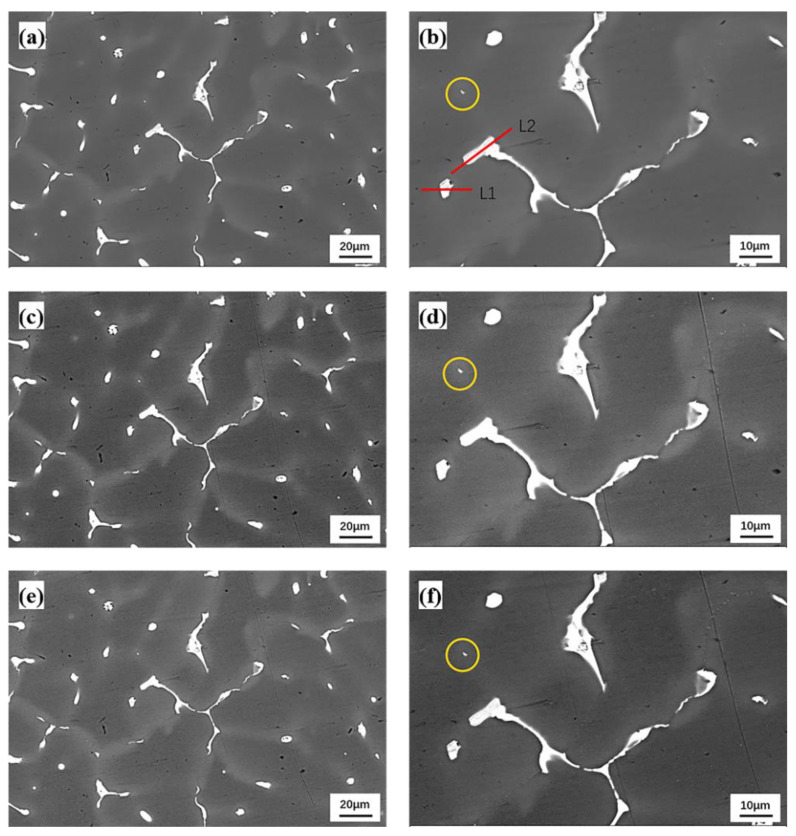
QISOZ backscattered SEM images at 500 times (**a**,**c**,**e**) and 1000 times (**b**,**d**,**f**) magnification at 100 °C (**a**,**b**), 200 °C (**c**,**d**) and 300 °C (**e**,**f**).

**Figure 6:**
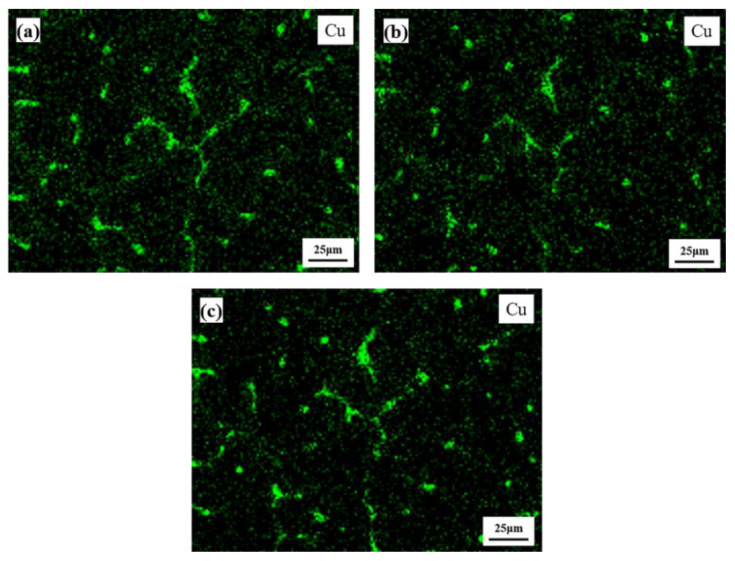
EDS elemental distribution of Cu with QISOZ magnification of 500 times at 100 °C (**a**), 200 °C (**b**) and 300 °C (**c**).

Figure 5 presents the backscattered SEM pictures of QISOZ, magnified by 500 times and 1000 times at 100 °C, 200 °C and 300 °C, where L1 and L2 in Figure 5b represent the line scan of the S phase mentioned in Figure 2d and Table 2. Comparing Figure 5a,c,e and Figure 5b,d,f, respectively, the size and morphology of the eutectic phases at 100 °C, 200 °C and 300 °C show no obvious changes, and even the very small eutectic phases circled in Figure 5b,d,f remained identical, and the gray zones of Cu-SZ showed no obvious changes in size and shape. Figure 6 shows the EDS elemental distribution of Cu with a QISOZ magnification of 500 times when the sample is heated to 100 °C, 200 °C and 300 °C. It can be seen that there is no obvious difference in the elemental distribution of Cu at these three temperatures. Cu is still slightly segregated in the gray region and heavily segregated in the eutectic phase. This shows that the diffusion of Cu is not obvious before 300 °C.

**Figure 7:**
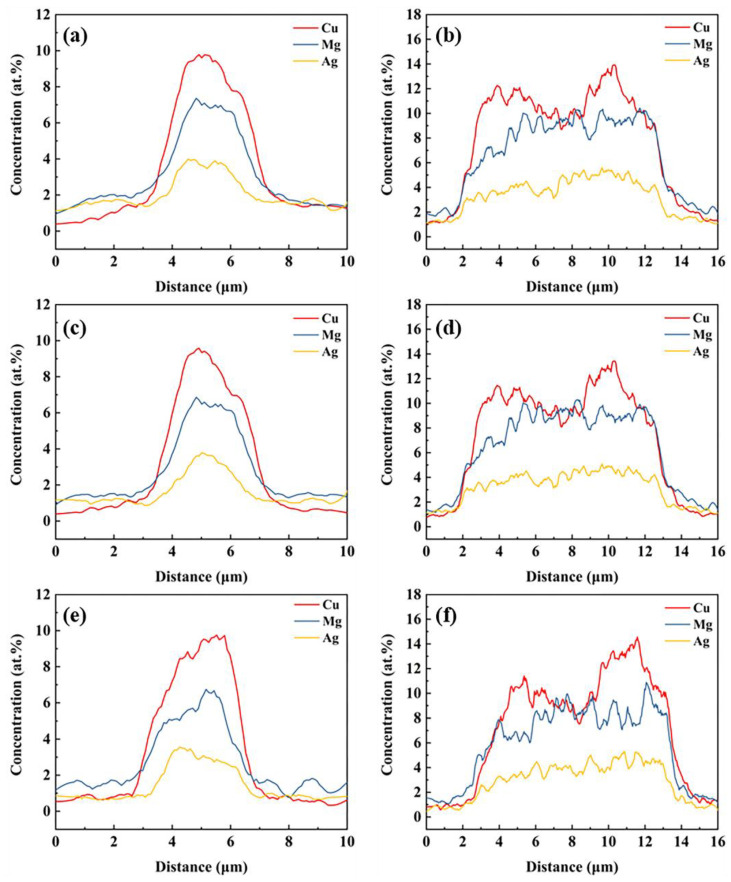
Elemental distribution along L1 (**a**,**c**,**e**) and L2 (**b**,**d**,**f**) at 100 °C (**a**,**b**), 200 °C (**c**,**d**) and 300 °C (**e**,**f**) of the sample.

Figure 7 shows the elemental distribution along L1 and L2 at 100 °C, 200 °C and 300 °C. The concentrations of Cu, Mg and Ag do not significantly change from 100 °C to 300 °C, the Cu/Mg is still close to 1:1, and slight segregation of Ag still exists in the S phase, demonstrating that the diffusion of Cu, Mg and Ag is still limited before reaching 300 °C.

The diffusion coefficient D follows the Arrhenius equation [40]:

(1) $$D=D_{0}\exp\left(-\frac{Q}{\mathrm{RT}}\right)$$

where D_0_ is the diffusion constant(m^2^/s), Q is the activation energy per mole of atoms(J/mol), T is the thermodynamic temperature(K), and R is the gas constant, which is 8.314 J/(mol· K).

According to Xie et al. [41], the diffusion constant of solute Cu and Mg in aluminum is 4.8 × 10^−5^ m^2^/s and 1.2 × 10^−4^ m^2^/s, respectively, and their diffusional activation energies are 133.6 × 10^3^ J/mol (Q_(Cu)_) and 131 × 10^3^ J/mol (Q_(Mg)_), respectively. Their diffusion coefficients at 300 °C can be calculated using Equation (1) (D_(Cu)_ = 1.92 × 10^−17^ m^2^/s, D_(Mg)_ =1.37 × 10^−16^ m^2^/s); such slow diffusion kinetics below 300 °C are consistent with the experimental observations.

### 3.3. Microstructural Characterization of the Sample at 400 °C, 450 °C and 500 °C

**Figure 8:**
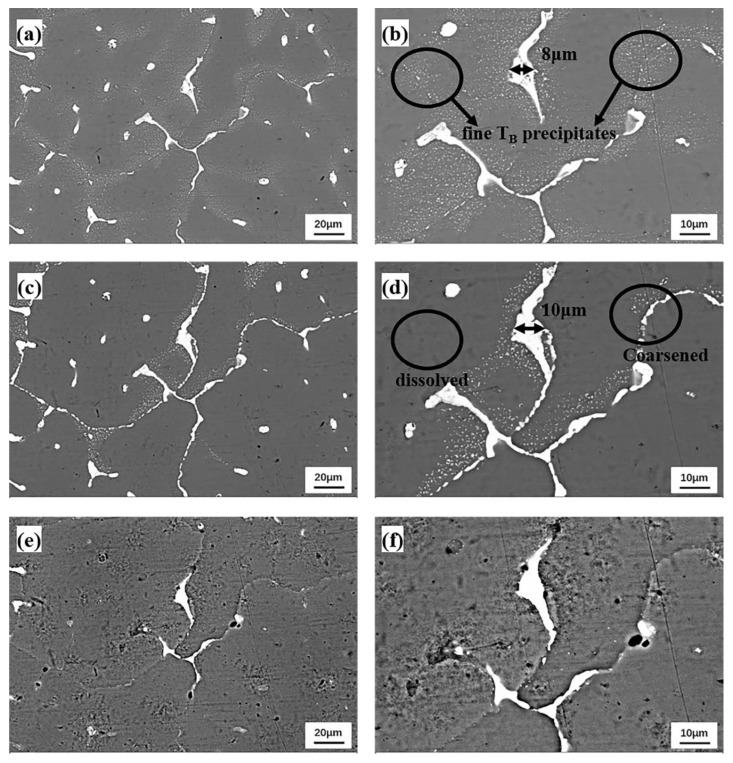
QISOZ backscattered SEM images at 500× (**a**,**c**,**e**) and 1000× (**b**,**d**,**f**) magnifications at 400 °C (**a**,**b**), 450 °C (**c**,**d**) and 500 °C (**e**,**f**).

**Figure 9:**
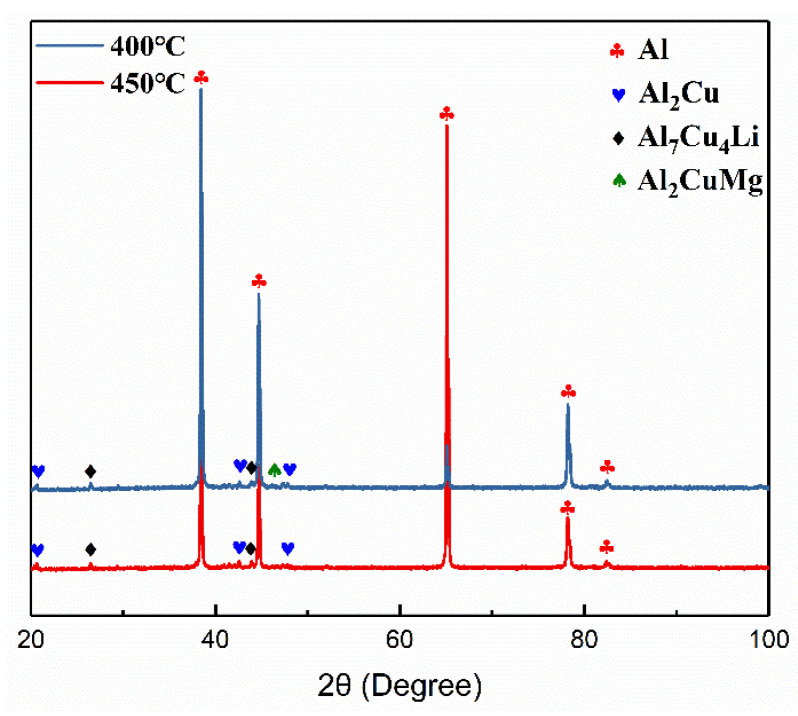
XRD pattern of the sample at 400 °C and 450 °C.

Figure 8 displays the backscattered SEM pictures of the sample at 400 °C, 450 °C and 500 °C, with QISOZ magnifications of 500 and 1000 times. Although the morphology of the eutectic phase does not significantly change at 400 °C, and Cu-SZ is still observed, small precipitates are noticed in the Cu-SZ for the first time. These small precipitates are probably Cu-containing phases (confirmed as T_B_ phases in Figure 9), as they are formed within the Cu-SZ. After further increasing the temperature to 450 °C, the Cu-SZ disappears, and the precipitates are roughened and uninterruptedly distributed on the grain boundary. The small precipitates that were previously observed in the grain interior are partially dissolved (circled in the picture), and the θ phases on the grain boundary are coarsened. This phenomenon can be elucidated by the Ostwald ripening theory [42,43,44,45]: because the fine T_B_ phases dissolve and the large T_B_ phases grow, the specific interface energy per unit quality decreases, and the total free energy of the system decreases. When the temperature reaches 500 °C, all the precipitates located on the grain boundaries are dissolved, and most of the small-sized eutectic phases dispersed within the grains are dissolved, leaving only large eutectic phases at the triangular grain boundary owing to their relatively slow dissolution speed. Additionally, some over-burnt pits can be found in the region with θ phases. This is because these samples were directly transferred to the furnace at 450 °C, and underwent subsequent heating from 450 °C to 500 °C over 50 min. It was reported [46] that when the temperature of the alloy rises rapidly, and beyond the melting point of the θ phase, θ phases will rapidly dissolve and leave obvious over-burnt pits.

Figure 9 shows the XRD result of the sample at 400 °C and 450 °C. When contrasted with the XRD result of the ingot at 400 °C, the diffraction peak of the T_B_ phase appears, and the peak strength of the S phase is weakened. At 450 °C, there is no diffraction peak of the S phase. Therefore, the fine precipitates observed at 400 °C are T_B_ phases, and the T_B_ phases coarsened at 450 °C.

**Figure 10:**
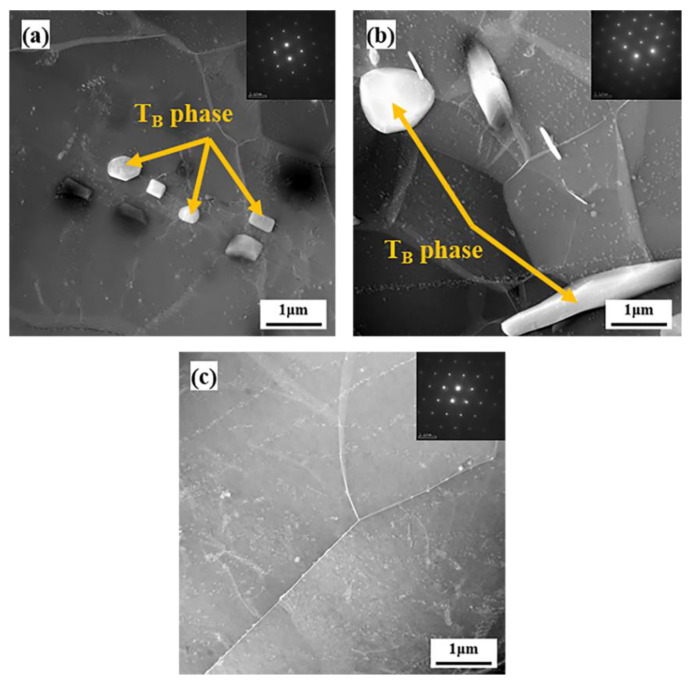
TEM images along <110>_Al_ zone axis of the samples at dissimilar temperatures: (**a**) 400 °C; (**b**) 450 °C; (**c**) 500 °C.

Figure 10 shows the TEM images along the <110>_Al_ zone axis of the sample heated to 400 °C, 450 °C and 500 °C. At 400 °C, fine T_B_ phases precipitate at and close to the grain boundary, and the T_B_ phases are coarsened at 450 °C. When the temperature increases to 500 °C, the T_B_ phases dissolve in the matrix. The TEM results are in keeping with the SEM results above.

**Figure 11:**
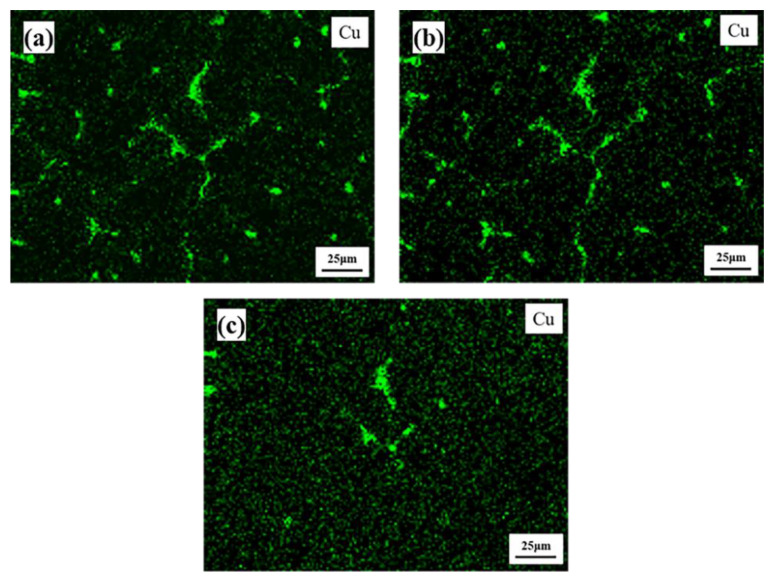
EDS elemental distribution of Cu with QISOZ magnification of 500 times at 400 °C (**a**), 450 °C (**b**) and 500 °C (**c**).

Figure 11 shows the EDS elemental distribution of Cu when the sample is heated to 400 °C, 450 °C and 500 °C. Cu becomes increasingly dispersed with elevated temperatures, and the θ phases on the grain boundary are first coarsened at 450 °C and then dissolved at 500 °C. This indicates that while the temperature was increased from 400 °C to 450 °C, the dissolution speed of the θ phase located on the grain boundary is less than the diffusion speed of Cu to the grain boundary in the Cu-SZ, and part of the Cu in the Cu-SZ directly diffuses into the grain, so the dispersion degree of Cu increases. The distribution of Cu at 500 °C is more dispersive than that at 450 °C because T_B_ phases and most of the θ phases are dissolved and increase the supersaturation of Cu in the alloy matrix.

**Figure 12:**
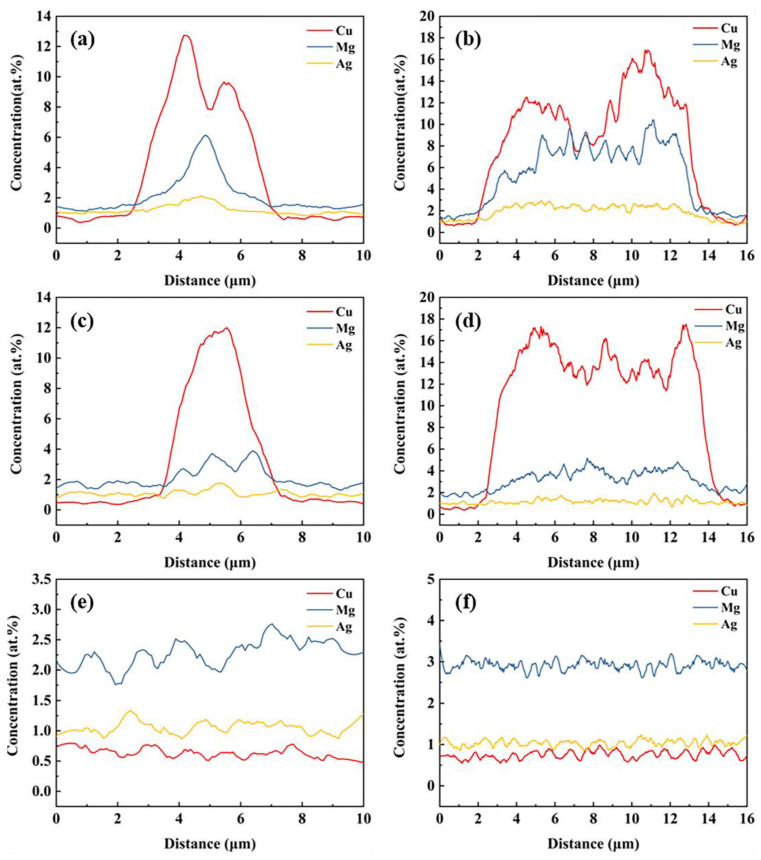
Elemental distributions along L1 (**a**,**c**,**e**) and L2 (**b**,**d**,**f**) at 400 °C (**a**,**b**), 450 °C (**c**,**d**) and 500 °C (**e**,**f**).

Figure 12 shows the elemental distributions along L1 and L2 at 400 °C, 450 °C and 500 °C. Compared with low-temperature data (see Figure 7e–f), Mg and Ag concentrations along L1 and L2 are decreased at 400 °C and the segregation of Ag disappears. When the temperature rises to 450 °C, the segregation of Mg along L1 and L2 is also removed, which is in keeping with the diminished XRD peaks in the S phases at 450 °C (see Figure 9). This suggests that diffusion of Ag and Mg from the S phase into the matrix is probably only activated above 300 °C and completed at 450 °C, transforming the S phase into the θ phase. Figure 12e,f show that the θ phases are dissolved when homogenizing at 500 °C.

At 400 °C, the diffusion coefficient of Mg in the Al matrix is 1.23 × 10^−14^ m^2^/s, which is nearly eight times faster than that of Cu (1.59 × 10^−15^ m^2^/s). Such a large discrepancy suggests that the diffusion of Mg from the S phase into the matrix occurs much more readily than Cu. Table 3 displays the dissolution temperature of S phases in Al-Cu-Li alloys that have dissimilar Cu/Mg ratios. When the Cu/Mg ratio increases, the dissolution temperature of the S phase decreases.

**Figure 13:**
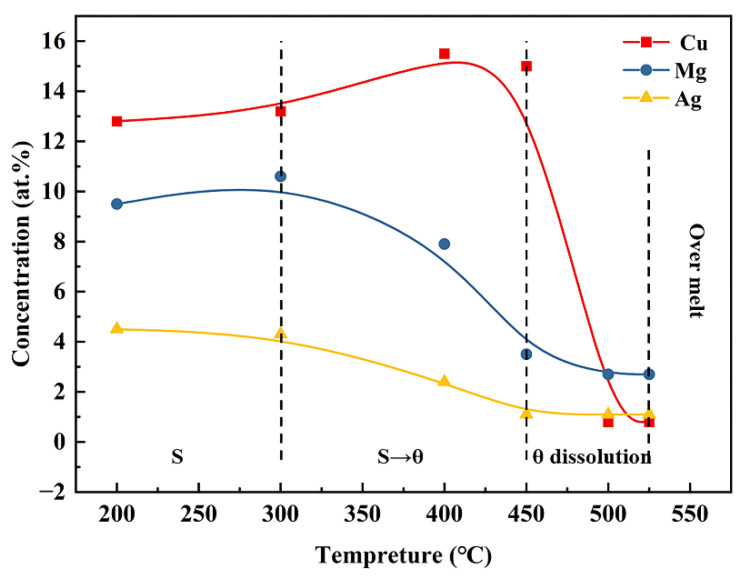
Evolution of elemental concentrations of Cu, Mg and Ag in S phase during homogenization.

Figure 13 summarizes the evolution of the elemental concentration in the S phase during homogenization. The concentrations of Cu, Mg and Ag show almost no changes before 300 °C; from 300 °C to 400 °C, Mg and Ag decrease while Cu slightly increases, suggesting that the S phase gradually transforms into the θ phase. Upon further increasing the temperature to 450 °C, the quantity of Cu starts to decrease as the S phase is continuously transformed into the θ phase and the θ phase is gradually dissolved within this temperature range; finally, from 450 °C to 500 °C, the transformed θ phase dissolves into the matrix, leading to a sharp decrease in Cu concentration.

**Figure 14:**
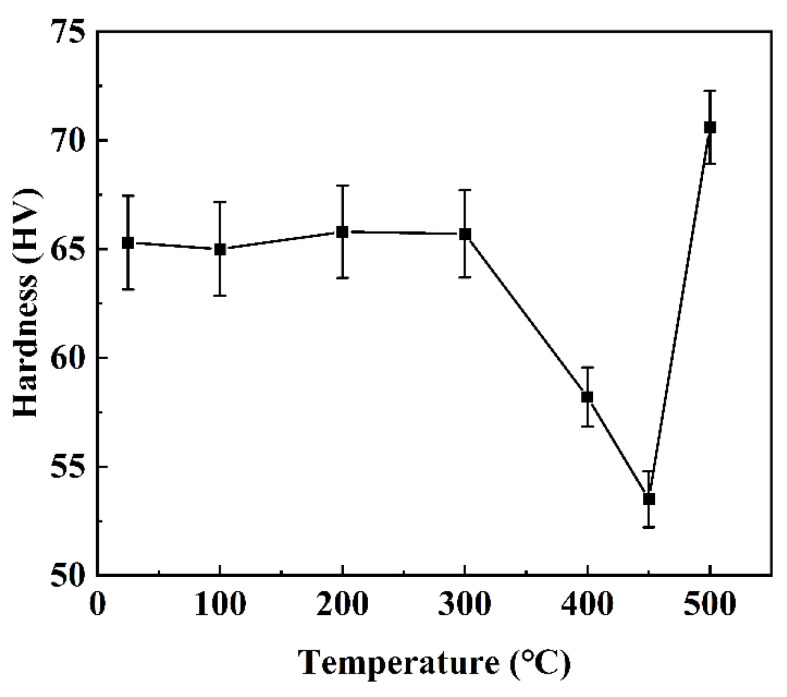
Evolution of sample hardness during homogenization.

Figure 14 shows the evolution of sample hardness during homogenization. Before 300 °C, the hardness barely changes at around 66 HV, which is consistent with the invariant phase configuration below 300 °C. When the temperature is improved from 300 °C to 450 °C, the hardness of the sample reduces from 66 HV to 54 HV, probably due to annealing-induced stress relief and recrystallization at this temperature [47]. The hardness of the sample rises from 54 HV to 71 HV when the temperature is increased to 500 °C, which is in keeping with our experimental observation (Figure 11) that most eutectic phases dissolve at this temperature and the supersaturation of Cu in the matrix has an apparent increase, leading to enhanced hardness.

**Figure 15:**
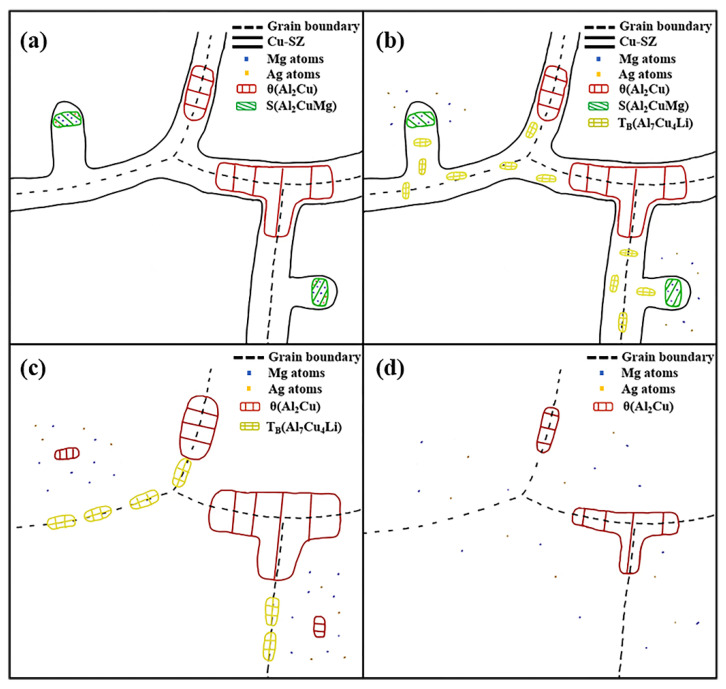
Schematic diagram of microstructural evolution during homogenization (**a**) 25–300 °C; (**b**) 400 °C; (**c**) 450 °C; (**d**) 500 °C.

Based on the above observations, Figure 15 illustrates the possible phase evolution process of the 2195 alloy during homogenization. As shown in Figure 15a, the microstructure of the sample does not alter when the temperature is elevated from 25 °C to 300 °C. When the temperature is improved to 400 °C (see Figure 15b), T_B_ phases precipitate from the Cu-SZ; this phenomenon was not found in previous studies. Ag and Mg in S phases diffuse into the matrix, and the segregation of Ag disappears. When further heated to 450 °C (see Figure 15c), the Cu-SZ disappears, the T_B_ phases and θ phases on the grain boundary are coarsened, Mg in the S phases continuously diffuses towards the matrix, transforming the S phase into the θ phase, and the newly formed θ phase starts to dissolve, which is consistent to the XRD results (see Figure 9) and aforementioned diffusivity calculation. Chen et al. [30] previously found that T_B_ and θ phases coarsened during the homogenization of the Al-5Cu-1Li-0.6Mg alloy, and Yang et al. [29] previously found that the S phase dissolved before the θ phase in the Al-3.52Cu-1.28Li-0.38Mg alloy. However, Chen did not distinguish the primary T_B_ eutectic phase from the later precipitated T_B_ phase, and neither of them found that the S phase transformed into the θ phase during homogenization. Some researchers have found the η-S phase transformation during the homogenization of the 7-series alloy (Al-Zn-Mg-Cu) by calculating the atomic diffusion velocity, SEM observation, TEM observation and other means [48,49], the S-θ phase transformation in this work could have similar behaviors. In further research, we will perform a more in-depth study on the S-θ phase transition, including the kinetics of such transformation and the effects of alloying elements and pretreatment parameters on this process, and apply computational simulations to study the microstructural evolution of Al-Cu-Li alloys. At 500 °C (see Figure 15d), the T_B_ phases located on the crystal boundary and the small-sized θ phases in the grain interior were all dissolved, and the large θ phases were partially dissolved, while the θ phases at the triangular crystal boundary dissolved more slowly.

## 4. Conclusions

In this paper, the evolution of the secondary phases (mainly the T_B_ phase and S phase), the main alloying elements and the mechanical properties of 2195 Al-Cu-Li alloys during homogenization were investigated. Cu-SZ was found at and near the grain boundary of 2195 ingot, and θ phases and S phases were distributed on Cu-SZ. When the sample was heated from 25 °C to 300 °C, the morphology and elemental distribution of the eutectic phase did not change, due to the insufficient diffusion kinetics at low temperatures. When the temperature was improved from 300 °C to 450 °C, the T_B_ phase first precipitated and then coarsened at the Cu-SZ due to Ostwald ripening, the Mg and Ag in the S phase diffused into the matrix, and the S phase was probably transformed into the θ phase. The hardness of the sample barely changed until heated above 300 °C. The sample was first softened when heating to 450 °C due to annealing, and then significantly increased from 450 °C to 500 °C due to the dissolution of most eutectic phases and enhanced solid-solution strengthening. The above results suggest that the homogenization temperature for the 2195 alloy can directly begin at 300 °C and should rapidly increase from 400 °C to 450 °C, avoiding the extension of the homogenization time owing to the coarsening of the θ and T_B_ phases on the grain boundaries. In the future, the phase evolution of 2195 Al-Cu-Li alloys will be investigated by means of computational simulations based on the experimental data.

## Figures and Tables

**Table 1:** Elemental ingredients of the 2195 ingot (wt. %).

Cu	Li	Mg	Ag	Zr	Zn	Mn	Fe	Si	Al
3.99	0.90	0.27	0.28	0.15	0.02	0.03	<0.02	<0.04	Bal.

**Table 2:** EDS results for each point in Figure 2d (at. %).

Point	Al	Cu	Mg	Ag	Closest Phase
A	68.8	30.6	0.38		Al_2_Cu
B	66.8	31.1	1.5		Al_2_Cu
C	71.6	13.2	10.6	4.5	Al_2_CuMg
D	78.7	10.7	7.1	3.4	Al_2_CuMg

**Table 3:** Effect of Cu/Mg ratio on the dissolution temperature of S phase.

	Cu/(wt. %)	Mg/(wt. %)	Cu/Mg	T _(S dissolve)_/°C
Li [27]	3.5	0.5	7	500
Yang [29]	3.52	0.38	9.26	495
Liu [26]	3.8	0.4	9.5	470
Present work	3.99	0.27	14.8	450

## Data Availability

Not applicable.
